# Sex-specific associations of Notch signaling with chronic HBV infection: a study from Taiwan Biobank

**DOI:** 10.1186/s13293-024-00641-z

**Published:** 2024-09-05

**Authors:** I-An Jen, Terry B. J. Kuo, Yung-Po Liaw

**Affiliations:** 1 Institute of Public Health, National Yang Ming Chiao Tung University, Taipei, Taiwan; 2 Institute of Brain Science, National Yang Ming Chiao Tung University, No. 155, Sec. 2, Li-Nong St., Beitou, Taipei, 11221 Taiwan; 3 Sleep Research Center, National Yang Ming Chiao Tung University, Taipei, Taiwan; 4 Department of Public Health and Institute of Public Health, Chung Shan Medical University, No. 110 Sec. 1 Jianguo N. Road, Taichung, 40201 Taiwan; 5 Department of Medical Imaging, Chung Shan Medical University Hospital, Taichung City, 40201 Taiwan

**Keywords:** Hepatitis B virus infection, Sex, Genetic variant, Notch signaling, POGLUT1, HIST1H2BC

## Abstract

**Background:**

Hepatitis B, a liver infection caused by the hepatitis B virus (HBV), can develop into a chronic infection that puts patients at high risk of death from cirrhosis and liver cancer. In this study, we aimed to investigate the difference of reactome pre-Notch expression and processing between males and females by using gene to function analysis in FUMA.

**Methods:**

We analyzed Taiwan Biobank (TWB) data pertaining to 48,874 women and 23,178 men individuals which were collected from 2008 to 2019. According to hepatitis B surface antigen (HBsAg) status in hematology, positive and negative were classified into case and control in the genome-wide association study (GWAS) analysis.

**Results:**

We found 4715 women and 2656 men HBV cases. The genomic risk loci were different between males and females. In male, three risk loci (rs3732421, rs1884575 and Affx-28516147) were detected while eight risk loci (Affx-4564106, rs932745, rs7574865, rs34050244, rs77041685, rs107822, rs2296651 and rs12599402) were found in female. In addition, sex also presented different results. In females, the most significant SNPs are gathered in chromosome 6. However, except for chromosome 6, significant HBV infection SNPs also could be found in chromosome 3 among males. We further investigated gene function in FUMA to identify the difference in reactome pre-Notch expression and processing between males and females. We found that POGLUT1 and HIST1H2BC only appeared in men but not in women.

**Conclusion:**

According to our study, the reactome pre-Notch expression including POGLUT1 and HIST1H2BC was associated with a risk of Hepatitis B in Taiwanese men when compared to women.

## Plain language summary

Hepatitis B is a serious liver infection caused by the hepatitis B virus (HBV). It can lead to long-term liver damage and cancer. We looked at differences in how the virus affects men and women in Taiwan. We analyzed data from over 72,000 people in the Taiwan Biobank. The study individuals were divided into two groups—those who had the hepatitis B virus (cases) and those who did not (controls). We looked for genetic differences between the two groups and found that the specific genetic risk factors for hepatitis B differed between men and women. We found three genetic risk factors in men and eight in women. This suggests that the way the hepatitis B virus interacts with our genes may differ between the sexes. We found that in women, the most significant genetic risk factors were all located on chromosome 6. However, in men, the significant risk factors were spread across different chromosomes, including chromosome 3. Finally, we looked at how these genetic differences might affect the way the body processes the hepatitis B virus. We found that two specific genes, called POGLUT1 and HIST1H2BC, were only linked to hepatitis B risk in men, not in women. This indicates that the biological pathways involved in hepatitis B infection may differ between males and females. Understanding these differences could lead to more effective, personalized treatment strategies for those affected by the virus.

## Highlights

- There are significant differences in genomic risk loci between male and female patients with hepatitis B virus (HBV) infection.
- Three risk loci (rs3732421, rs1884575 and Affx-28516147) in male were detected while eight risk loci (Affx-4564106, rs932745, rs7574865, rs34050244, rs77041685, rs107822, rs2296651 and rs12599402) were found in female.
- Investigated gene function in FUMA to identify the difference of reactome pre-Notch expression and processing between males and females. We found that POGLUT1 and HIST1H2BC only appeared in males but not in females.

## Background

Hepatitis B virus (HBV) infection is one of the major risk factors for advanced liver diseases worldwide. Individuals with HBV infection can develop a chronic infection that puts patients at high risk of death from cirrhosis and hepatocellular carcinoma [1]. Although mass immunization programs recommended by the World Health Organization since 1991 have greatly reduced HBV infection rates in infants, children, and adolescents in many countries. However, not all countries followed these recommendations, and large numbers of people were still infected with HBV before immunization programs were implemented. There is an urgent need to understand how to manage chronic HBV infection to reduce the morbidity and mortality of chronic HBV infection [2].

The male preference in viral gene expression/replication and disease progression was demonstrated in the animal study [3]. Clinical studies of hepatitis B around the world also support that chronic hepatitis B appears to progress more rapidly in men than in women. This may be related to sex hormones that bind to their specific cell receptors and affect the corresponding signaling pathways, regulate the transactivation of HBV X virus protein (HBx), cause the chronic release of inflammatory cytokines in the hepatocyte microenvironment, and further participate in hepatocytes. All these effects may be related to sex disparity in the occurrence and progression of hepatitis B virus-associated hepatocellular carcinoma (HCC) [4]. Although Human Leukocyte Antigen class II variants have been reported to be associated with the development of chronic hepatitis after HBV infection [5]. However, the pathogenic mechanism of the development of chronic hepatitis after HBV infection, especially the sex-specific regulation of gene expression in liver and extrahepatic tissues, remains poorly understood. Studies have reported that the androgen axis plays a tumor-promoting role in hepatocarcinogenesis, and the estrogen axis has a tumor-inhibiting role, but in some cases, the androgen pathway can act in the opposite way, and the estrogen pathway also has the same effects [6]. It can be seen that the influence factor of sex on the development of chronic hepatitis from HBV infection is not just because of sex hormones.

The Notch signaling pathway is a highly conserved cell signaling system that exists in most animals. Through a direct pathway from the membrane to the nucleus, the Notch pathway promotes tissue growth and carcinogenesis in some cases, but cell death and tumor suppression in others [7]. Abnormal activation or inactivation of Notch signaling pathway can lead to human disease, including many different cancer types [8]. Notch signaling plays an important role in both normal liver development and liver tumorigenesis [9]. Abnormalities of Notch-related genes are associated with the aggressiveness of liver cancer [10]. Studies have demonstrated that Notch signaling regulates the HCC tumor microenvironment, tumorigenesis, progression, angiogenesis, invasion, and metastasis, and is associated with HCC development and progression [11]. In addition, both in vitro and in vivo experiments found that HBx may through activate the Notch signaling pathway to promote the growth of human non-tumor hepatocyte cell line L02 cells and the progression of HCC [12, 13]. However, there are no study to investigate the role of reactome pre-Notch expression and processing in chronic HBV infection between men and women. In this study, we used the large-scale GWAS carried out in a HBV highly endemic area aimed to explore sex-specific genetic variants associated with chronic HBV infection.

## Methods

### Taiwan Biobank

Data in the current study were obtained from Taiwan Biobank which was collected from 2008 to 2019. Taiwan Biobank is a prospective cohort database which contained genetic and clinical data. Volunteers with no cancer history, within 30 to 70 years old and surely a Taiwanese could enter the database in recruitment station. Approval for this study was provided by the Institutional Review Board of Chung Shan Medical University (IRB: CS1-23101). Before the enrollment, participants had to sign inform consent.

### Gene data of GWAS analysis

Gene data in the analysis were from Taiwan Biobank. In this study, all 73,196 people were tested with the TWBv2.0 chip. The chip of single nucleotide polymorphisms (SNPs) was TWBv2.0 from Affymetrix using the Axiom™ Genome-Wide Array Plate System (Affymetrix, Santa Clara, CA, USA). The quality control of SNP reached criteria below would be excluded: (1) Hardy–Weinberg equilibrium test < 0.000001; (2) minor allele frequency < 0.001; (3) call rate < 5%. A total of 73,196 subjects and 489,276 SNPs finally in the gene dataset.

### Definition of HBV

Data of HBV were from Taiwan Biobank. According to hepatitis B surface antigen (HBsAg) status in hematology. Equivocal or weakly of HBsAg would be excluded from analysis (N = 803). Moreover, positive and negative was classified into case and control in the GWAS analysis.

### GWAS and FUMA GWAS analysis

PLINK version1.9 was used to do GWAS analysis while SAS 9.4 software was used to classify covariates. Model in GWAS was adjusted for age and five principal components. Significant threshold was 5e-8 in the current GWAS analysis.

FUMA GWAS is a platform that can do the annotation, visualization, and so on after the GWAS analysis (https://fuma.ctglab.nl/). We used FUMA GWAS to find out the genomic risk loci analysis in both men and women, draw the Manhattan plot and also the pre notch expression and processing.

### Statistical analysis

The analysis in Table were perform by ANOVA and Student’s t-test. ANOVA was used to compare the differences of gender, HBV infection and continuous variables (age and BMI) and presented by mean (± standard error). Furthermore, the differences of gender, HBV infection and categories were examined by Student’s t-test which were showed by number and percentage (%).

## Results

Table 1 showed the results of basic characteristics of participants among gender, HBV infection status and confounders. There were 23,178 men in the GWAS study, including 2656 HBV cases and 20,522 with no HBV infection subjects. Moreover, there were 48,874 women in the analysis which contained 4715 HBV cases and 44,159 healthy control subjects. HBV infection was significant between gender (P-value < 0.0001). All the covariates reached statistical significant difference between gender, including age (P-value = 0.0006), exercise (P-value < 0.0001), cigarette smoking (P-value < 0.0001), alcohol drinking (P-value < 0.0001), body mass index (P-value < 0.0001), hypertension (P-value < 0.0001), hyperlipidemia (P-value < 0.0001) and diabetes (P-value < 0.0001). In addition, all the variables were significant among genders with or without HBV infection. In age, mean age was 50.407 (± 0.079) years in male without HBV, 50.000 (± 0.194) years in male with HBV, 50.063 (± 0.050) years in female without HBV and 50.041 (± 0.136) years in female with HBV. The P-value of age was 0.0013. In exercise, a total of 11,651 (56.77%) no exercise and 8871 (43.23%) exercise in male without no HBV, 1613 (60.73%) no exercise and 1043 (39.27%) exercise in male with HBV. However, 26,665 (60.38%) no exercise and 17,494 (39.62%) exercise subjects were in female without HBV, 2885 (61.19%) no exercise and 1830 (38.81%) exercise in female with HBV. The P-value was < 0.0001.

**Table 1:** Basic characteristics of participants stratified by gender and HBV infection status

Variables	Male without HBV	Male with HBV	Female without HBV	Female with HBV	*P*-value
Age, years	50.407 ± 0.079	50.000 ± 0.194	50.063 ± 0.050	50.041 ± 0.136	**0.0013**
Exercise, n %					**< 0.0001**
No	11,651 (56.77)	1613 (60.73)	26,665 (60.38)	2885 (61.19)	
Yes	8871 (43.23)	1043 (39.27)	17,494 (39.62)	1830 (38.81)	
Cigarette smoking, n %					**< 0.0001**
No	11,138 (54.27)	1469 (55.31)	41,595 (94.19)	4456 (94.51)	
Yes	9384 (45.73)	1187 (44.69)	2564 (5.81)	259 (5.49)	
Alcohol drinking, n %					**< 0.0001**
No	16,584 (80.81)	2163 (81.44)	42,918 (97.19)	4584 (97.22)	
Yes	3938 (19.19)	493 (18.56)	1241 (2.81)	131 (2.78)	
Body mass index, kg/m^2^	25.487 ± 0.025	25.229 ± 0.068	23.603 ± 0.018	23.515 ± 0.055	**< 0.0001**
Hypertension, n %					**< 0.0001**
No	16,889 (82.30)	2229 (83.92)	39,896 (90.35)	4322 (91.66)	
Yes	3633 (17.70)	427 (16.08)	4263 (9.65)	393 (8.34)	
Hyperlipidemia, n %					**< 0.0001**
No	18,463 (89.97)	2447 (92.13)	41,253 (93.42)	4467 (94.74)	
Yes	2059 (10.03)	209 (7.87)	2906 (6.58)	248 (5.26)	
Diabetes, n %					**< 0.0001**
No	19,024 (92.70)	2513 (94.62)	42,241 (95.66)	4552 (96.54)	
Yes	1498 (28.48)	143 (5.38)	1918 (4.34)	163 (3.46)	

Bold indicates statistical significance

n: sample size; %: percent; BMI: body mass index; kg: kilogram; m^2^: meter squared

In Table 2, genomic risk loci were different between men and women. In male, three risk loci (rs3732421, rs1884575 and Affx-28516147) were detected while eight risk loci (Affx-4564106, rs932745, rs7574865, rs34050244, rs77041685, rs107822, rs2296651 and rs12599402) were found in female.

**Table 2:** Genomic risk loci of HBV infection in men and women

Genomic locus	uniqID	rsID	chr	pos	P-value	Start	End
Male							
1	3:119150089:A: G	rs3732421	3	119,150,089	4.8E–08	119,111,870	119,252,208
2	6:25654089:C: G	rs1884575	6	25,654,089	1.03E–13	25,331,300	29,613,163
3	6:33179689:C: T	Affx-28,516,147	6	33,179,689	1.05E–12	33,173,842	33,197,589
Female							
1	1:1152631:A: C	Affx-4,564,106	1	1,152,631	1.76E–08	1,123,434	1,153,113
2	1:160428832:A: G	rs932745	1	160,428,832	3.40E–11	160,407,025	160,468,756
3	2:191964633:G: T	rs7574865	2	191,964,633	1.09E–08	191,925,424	191,970,120
4	2:204696941:A: G	rs34050244	2	204,696,941	1.31E–09	204,690,355	204,758,358
5	6:29551737:C: T	rs77041685	6	29,551,737	3.50E–18	25,331,300	29,613,163
6	6:33175575:C: T	rs107822	6	33,175,575	1.72E–15	33,173,842	33,570,932
7	14:70245193:A: G	rs2296651	14	70,245,193	5.83E–11	70,245,193	70,245,193
8	16:11189888:C: T	rs12599402	16	11,189,888	4.76E–09	11,154,770	11,201,428

In Table 3, the differences in SNPs genotypes stratified by gender and HBV infection were presented. There were 11 SNPs significant among males or females in HBV infection in GWAS analysis. In rs3732421, there were 9217 (44.95%) AA, 8974 (43.76%) AG and 2314 (11.29%) in male without no HBV, 1048 (39.49%) AA, 1252 (47.17%) AG and 354 (13.34%) GG in male with HBV. Moreover, there were 19,644 (44.54%) AA, 19,595 (44.43%) AG and 4869 (11.04%) AG in females without HBV, 1984 (42.12%) AA, 2148 (45.61%) AG and 578 (12.27) GG in female with HBV. The P-value of the difference was < 0.0001. The only one SNP which was not significant was rs77041685 (P-value = 0.6100). There were 17,499 (85.33%) CC, 2894 (14.11%) CT and 115 (0.56%) TT in male without no HBV, 2254 (84.86%) CC, 387 (14.57%) CT and 15 (0.56%) in male with HBV. Moreover, there were 37,378 (84.71%) CC, 6479 (14.68%) CT and 268 (0.61%) in female without HBV and 3996 (84.84%) CC, 684 (14.52%) CT and 30 (0.64%) TT in female with HBV.

**Table 3:** Frequencies of HBV related SNPs stratified by gender and HBV infection status

Variables	Male without HBV	Male with HBV	Female without HBV	Female with HBV	*P*-value
rs3732421					< 0.0001
AA	9217 (44.95)	1048 (39.49)	19,644 (44.54)	1984 (42.12)	
AG	8974 (43.76)	1252 (47.17)	19,595 (44.43)	2148 (45.61)	
GG	2314 (11.29)	354 (13.34)	4869 (11.04)	578 (12.27)	
rs1884575					0.0053
CC	17,852 (87.22)	2265 (85.50)	37,905 (86.10)	4077 (86.71)	
CG	2526 (12.34)	369 (13.93)	5924 (13.46)	607 (12.91)	
GG	89 (0.43)	15 (0.57)	194 (0.44)	18 (0.38)	
Affx-28,516,147					< 0.0001
CC	14,437 (70.43)	2083 (78.51)	31,224 (70.78)	3609 (76.61)	
CT	5554 (27.10)	541 (20.39)	11,835 (26.83)	1029 (21.84)	
TT	507 (2.47)	29 (1.09)	1053 (2.39)	73 (1.55)	
Affx-4,564,106					< 0.0001
CC	11,634 (56.80)	1419 (53.49)	25,246 (57.26)	2515 (53.43)	
CA	7662 (37.41)	1038 (39.13)	16,245 (36.84)	1867 (39.66)	
AA	1187 (5.80)	196 (7.39)	2602 (5.90)	325 (6.90)	
rs932745					< 0.0001
GG	13,431 (65.58)	1790 (67.52)	28,801 (65.32)	3309 (70.25)	
GA	6333 (30.92)	766 (28.89)	13,721 (31.12)	1275 (27.07)	
AA	716 (3.50)	95 (3.58)	1569 (3.56)	126 (2.68)	
rs7574865					< 0.0001
TT	8465 (41.37)	1193 (45.05)	18,309 (41.62)	2130 (45.27)	
TG	9416 (46.01)	1164 (43.96)	20,164 (45.84)	2086 (44.34)	
GG	2582 (12.62)	291 (10.99)	5518 (12.54)	489 (10.39)	
rs34050244					< 0.0001
GG	12,667 (61.79)	1583 (59.69)	27,579 (62.52)	2749 (58.37)	
GA	6889 (33.60)	939 (35.41)	14,593 (33.08)	1683 (35.73)	
AA	945 (4.61)	130 (4.90)	1940 (4.40)	278 (5.90)	
rs77041685					0.6100
CC	17,499 (85.33)	2254 (84.86)	37,378 (84.71)	3996 (84.84)	
CT	2894 (14.11)	387 (14.57)	6479 (14.68)	684 (14.52)	
TT	115 (0.56)	15 (0.56)	268 (0.61)	30 (0.64)	
rs107822					< 0.0001
CC	8771 (42.79)	1324 (49.94)	18,902 (42.84)	2370 (50.32)	
CT	9321 (45.47)	1121 (42.29)	19,936 (45.18)	1950 (41.40)	
TT	2405 (11.73)	206 (7.77)	5289 (11.99)	390 (8.28)	
rs2296651					< 0.0001
GG	16,685 (81.31)	2190 (82.49)	35,922 (81.35)	3960 (83.99)	
GA	3570 (17.40)	464 (17.48)	7726 (17.50)	755 (16.01)	
AA	265 (1.29)	1 (0.04)	510 (1.15)	0 (0.00)	
rs12599402					< 0.0001
TT	7084 (34.56)	814 (30.73)	15,178 (34.42)	1438 (30.52)	
TC	9956 (48.58)	1355 (51.15)	21,309 (48.32)	2351 (49.90)	
CC	3456 (16.86)	480 (18.12)	7615 (17.27)	922 (19.57)	

**Fig. 1:**
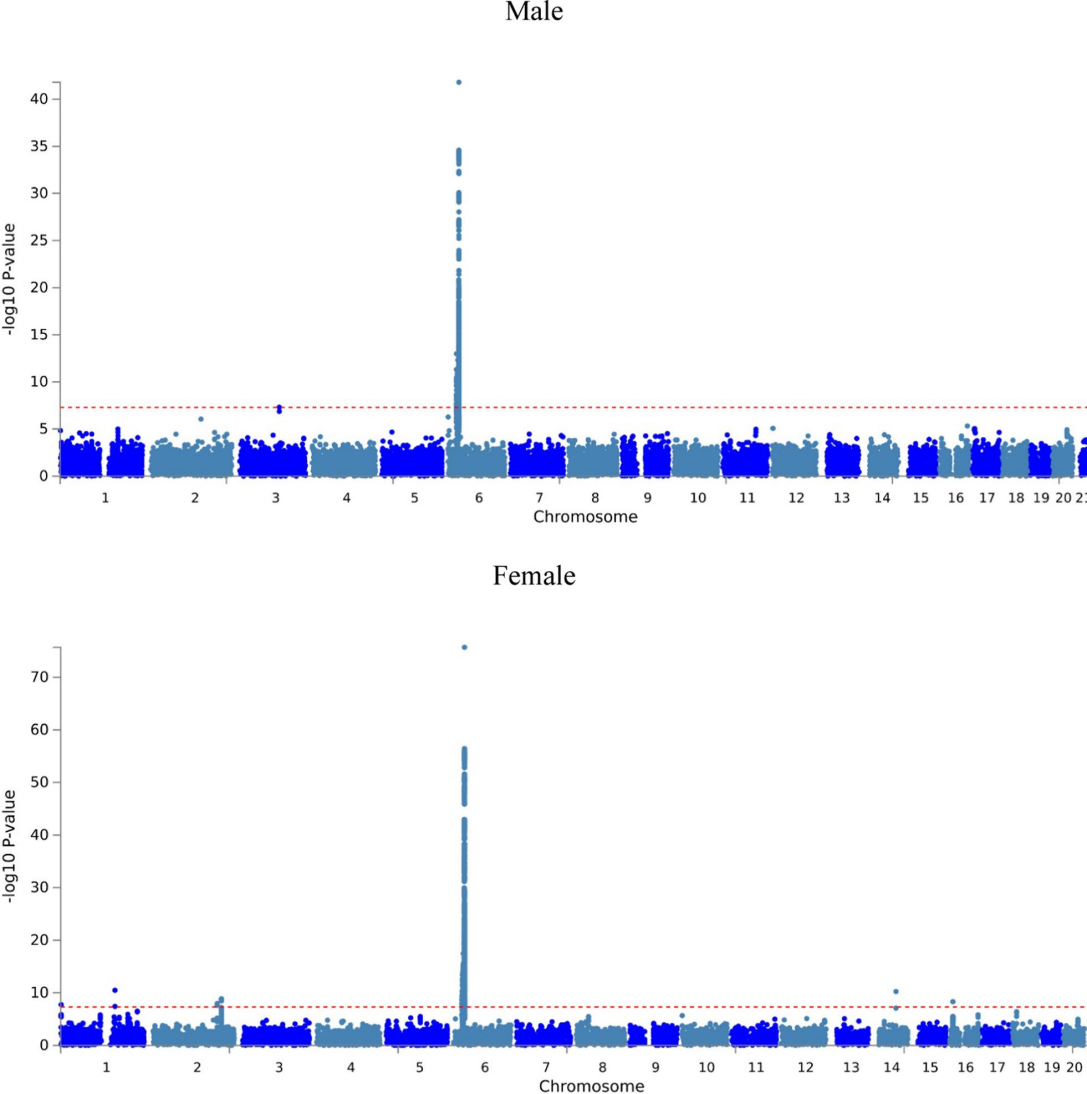
Manhattan plot of HBV infection in men and women

In Manhattan plot (Fig. 1), sex also presented different result. In females, the most significant SNPs gathered in chromosome 6. However, excepted for chromosome 6, significant HBV infection SNPs also could be found in chromosome 3 among males.

Table 4 shows the results of gene to function analysis in FUMA and want to know the difference of reactome pre-Notch expression and processing between men and women. We found that POGLUT1 and HIST1H2BC only appeared in males but not in females.

**Table 4:** Result of Reactome pre notch expression and processing of HBV in men and women

Male	Female
POGLUT1, HIST1H3A, HIST1H4A, HIST1H4B, HIST1H2AB, HIST1H2BB, HIST1H4C, HIST1H2BC, HIST1H2AC, HIST1H2BD, HIST1H2BE, HIST1H4D, HIST1H3D, HIST1H2AD, HIST1H2BF, HIST1H4E, HIST1H2BG, HIST1H2AE, HIST1H3E, HIST1H4F, HIST1H3F, HIST1H2BH, HIST1H3G, HIST1H2BI, HIST1H4H, HIST1H2BJ, HIST1H2BK, HIST1H4I, HIST1H2BL, HIST1H3H, HIST1H2AJ, HIST1H2BM, HIST1H4J, HIST1H4K, HIST1H2BN, HIST1H3I, HIST1H4L, HIST1H3J, HIST1H2BO	HIST1H3A, HIST1H4A, HIST1H4B, HIST1H2AB, HIST1H2BB, HIST1H4C, HIST1H2AC, HIST1H2BD, HIST1H2BE, HIST1H4D, HIST1H3D, HIST1H2AD, HIST1H2BF, HIST1H4E, HIST1H2BG, HIST1H2AE, HIST1H3E, HIST1H4F, HIST1H3F, HIST1H2BH, HIST1H3G, HIST1H2BI, HIST1H4H, HIST1H2BJ, HIST1H2BK, HIST1H4I, HIST1H2BL, HIST1H3H, HIST1H2AJ, HIST1H2BM, HIST1H4J, HIST1H4K, HIST1H2BN, HIST1H3I, HIST1H4L, HIST1H3J, HIST1H2BO
*Only appear in males*	
POGLUT1, HIST1H2BC	

## Discussion

Chronic HBV infection is a condition that affects more than 240 million individuals worldwide and can lead to cirrhosis, liver failure, and liver cancer [14]. Chronic HBV infection is a critical risk factor for HCC development. The sex disparity of HBV-related HCC has been noticed for a long time [15]. In Taiwan, epidemiological observations revealed that the incidence of HBV-related HCC was much higher in men than in women. Studies suggested that male gender was a significant risk factor in HBV-induced HCC [16, 17]. The influence of sex on HBV-induced HCC begins at a relatively early stage in chronic hepatitis B by modulating many host factors, including the levels of sex hormones and immune responses and epigenetic and genetic alternations [15]. In this study, we found 4715 females and 2656 males HBV cases in Taiwan Biobank data and collected from 2008 to 2019. We found the genomic risk loci were different between men and women. There are three risk loci (rs3732421, rs1884575 and Affx-28516147) were detected in men and eight risk loci (Affx-4564106, rs932745, rs7574865, rs34050244, rs77041685, rs107822, rs2296651 and rs12599402) were detected in women. Among them, the most prominent SNPs for females clustered on chromosome 6. However, in addition to chromosome 6, significant HBV infection SNPs were also found on chromosome 3 in males. We further investigated the gene function in FUMA to determine the differences in reactome pre-Notch expression and processing between men and women. We found that POGLUT1 and HIST1H2BC are only present in men but not in women.

POGLUT1, a newly identified gene within a decade that contains 11 exons and encodes a protein with 392 amino acids, has orthologs across multiple species. POGLUT1 has both glucosyltransferase and xylosyltransferase functions, with variable effects on cellular proliferation under different conditions [18]. The first human disease associated with POGLUT1 pathogenic variants was Dowling-Degos disease. Dowling-Degos disease is an autosomal dominant dermatosis characterized by progressive reticulate hyperpigmentation [19]. Subsequent studies found that POGLUT1 glycosylates the extracellular domain of Notch receptors associated with muscle disease and adds a new dimension to the relationship between Notch signaling and skeletal muscle that suggested biallelic pathogenic variants in POGLUT1 related to muscular dystrophy [20]. To date, there is no literature reporting the correlation between chronic HBV infection and POGLUT1. However, studies in mammalian cells had identified that POGLUT1 is an essential regulator of Notch signaling [21]. Notch signaling is a pathway implicated in the maintenance of stem cells, cell fate specification, proliferation, apoptosis, and immune responses during embryogenesis and in self-renewing tissues of the adult organism [22]. There is growing evidence suggested that Notch signaling contribute to liver inflammation in HBV infection [23]. In acute HBV infection, activation of Notch signaling triggered excessive production of regulatory T cells (Tregs), which in turn suppresses CD4/CD8 T cells, to cause the decrease of responsiveness of T cells, therefore reduced the clearance of HBV. During HBV infections, the increase in Tregs is inversely proportional to HBV DNA levels. Paradoxically, the induction of Tregs also leads to a decrease in virus-specific T cell responses. Both circulating and intratumoral Tregs further promote the development of HBV-related HCC by impairing the functions of CD8 T cells [24]. However, during chronic HBV infection, there is a decline in the expression of Notch signaling. Still, as cirrhosis and HCC develop, the expression of Notch signaling is once again increased [25]. Therefore, it is speculated that the Notch signaling may be involved in the progression of HBV-related HCC. Studies have unveiled the multifaceted involvement of the Notch signaling in various stages of HBV-related HCC development [26–28]. Notch activation enhanced intratumoral fibrosis in cholangiocarcinoma-like-HCC and is associated with poor clinical outcomes [28]. However, the role of Notch signaling in sex-specific associations with chronic HBV infection has not been reported.

HIST1H2BC (histone cluster 1 H2B family member c) is a gene encoding a protein that serves as the core component of the nucleosome. This protein plays an important role in transcriptional regulation, DNA replication, and chromosome stability. HIST1H2BC-produced protein can act as an antimicrobial peptide [29] and play an important role in inflammation or immune evasion [30, 31]. Studies have shown that HIST1H2BC has the highest alteration rate in breast invasive ductal carcinoma [30], diffuse large B-cell lymphoma, focal lymphoma, colon adenocarcinoma, and colorectal adenocarcinoma. However, no study has reported the association between chronic HBV infection and HIST1H2BC.

In conclusion, this study is the first to identify that POGLUT1 and HIST1H2BC only appeared in males, not in females, and were associated with the risk of Hepatitis B in Taiwanese individuals. Further studies are needed to confirm the roles of POGLUT1 and HIST1H2BC in HBV-related HCC.

## Data Availability

The data that support the findings of this study are available from Taiwan Biobank but restrictions apply to the availability of these data, which were used under license for the current study, and so are not publicly available. Data are however available from the authors upon reasonable request and with permission of Taiwan Biobank.

## Abbreviations

HBV: Hepatitis B virus
HBsAg: Hepatitis B surface antigen
chr: Chromosome
HCC: Hepatocarcinoma
HIST1H2BC: Histone cluster 1 H2B family member c
GWAS: Genome-wide association studies
SNP: Single nucleotide polymorphism
